# Serum TSH level as predictor of Graves’ disease recurrence following antithyroid drug withdrawal: A systematic review

**DOI:** 10.1371/journal.pone.0245978

**Published:** 2021-01-29

**Authors:** Imam Subekti, Gracia Jovita Kartiko, Zahra Farhanni Suhardi, Muhadi, Wismandari Wisnu

**Affiliations:** 1 Faculty of Medicine, Division of Endocrinology and Metabolism, Department of Internal Medicine, Dr. Cipto Mangunkusumo General Hospital, Universitas Indonesia, Jakarta, Indonesia; 2 Faculty of Medicine, Department of Internal Medicine, Dr. Cipto Mangunkusumo General Hospital, Universitas Indonesia, Jakarta, Indonesia; 3 Faculty of Medicine, Division of Cardiology, Department of Internal Medicine, Dr. Cipto Mangunkusumo General Hospital, Universitas Indonesia, Jakarta, Indonesia; Zagazig University, EGYPT

## Abstract

Graves’ disease (GD) has a high recurrence rate despite various and adequate treatment. Numerous studies have been performed to identify the predictor of disease recurrence. This report aims to investigate the role of thyroid stimulating hormone (TSH) level as a thyrotropin in predicting the recurrence of Graves’ disease within 1 to 2 years following antithyroid drug (ATD) withdrawal. Literature searching was conducted on PubMed, Scopus, Cochrane, Proquest, EBSCO in August 2019 and Google Scholar in October 2020. The study criteria include the study that evaluates TSH level 4 weeks following ATD withdrawal, with subjects ≥18 years old who are retrospectively or prospectively followed up after 1 to 2 years following ATD withdrawal. Four eligible studies were selected based on inclusion/exclusion criteria, all of which measured TSH level at 4 weeks following ATD withdrawal. All studies had 1 to 2 years follow up. One study was an RCT, two studies were done in prospective cohort and another in retrospective cohort. All studies had comparable validity and applicability. Three out of four studies suggested that low TSH level measured 4 weeks following treatment withdrawal was associated with higher risk of disease recurrence. In conclusion, low TSH level obtained 4 weeks after ATD withdrawal was associated with higher rate of recurrence rate in GD.

## Introduction

Graves’ disease (GD) is the most common autoimmune disorder with an incidence of 0.5% in the population [1–5]. In addition, GD is one of the most common causes of hyperthyroidism which accounts for 50–80% of this disorder besides toxic nodular goiter (TNG) [3–8]. According to Indonesian National Health Survey in 2013, the prevalence of hyperthyroidism based on history taking was 0.4% and was found to be higher in women, urban population, and above 45 years old [9]. While in the US, GD prevalence is slightly higher with approximately 1.2% of its population, consisting 0.5% overt hyperthyroidism and 0.7% subclinical hyperthyroidism [10].

There are three main modalities in the management of GD, namely antithyroid drugs (ATDs), radioactive iodine (RAI), and thyroidectomy with β-blockers therapy as the additional therapy for ameliorating the symptoms of GD [6,7,10]. Antithyroid drugs are the first line treatment and usually requires 12 to 18 months to achieve euthyroidism in most cases [1,6,7,10,11]. However, the rate of recurrence of GD remains high, ranging from 37–50% in many studies [12–14].

Numerous studies have been done to identify factors influencing the recurrence of GD, such as age, gender, thyroid size, antithyroid dosage, smoking history, thyroid echogenicity, thyroid volume, serum TSH level, thyrotropin receptor antibody (TRAb), and thyroxin peroxidase antibody (TPO-Ab) [15–29]. A meta-analysis of pre-treatment GD patients found some significant risk factors associated with disease recurrence following ATD withdrawal. Those risk factors included the presence of orbitopathy, smoking, larger thyroid volume and biochemically more severe disease assessed from free T4 (fT4), total T3 (tT3), TRAb, thyrotropin binding inhibiting immunoglobulin (TBII) and thyrotropin stimulating antibody (TSAb) levels [14]. Graves’ Recurrent Events After Therapy (GREAT) Score has been developed by Vos et al. in the Netherlands [12]. The parameters included in the score calculation were age, fT4, TBII, goiter size and genetical examination for GREAT + Score. However, due to high cost and low availability of TBII, TSAb and genotyping examination, the score is unfavorable to be used in developing countries, such as Indonesia.

The TSH level after ATDs withdrawal showed promising value in predicting GD recurrence [17,18,26]. Measurement of TSH level is practical, affordable, and widely available in daily practice. In this systematic review, we aim to systematically search for the evidence of TSH level in predicting the recurrence of GD following treatment withdrawal.

## Materials and methods

The protocol of this systematic review has been registered in International Prospective Register for Systematic Reviews (PROSPERO) with registration number CRD42020147750. This systematic review also adheres Preferred Reporting Items for Systematic Reviews and Meta-Analysis (PRISMA) guidelines [30].

The characteristic of this study is described using patient, indicator/intervention, comparison, outcome (PICO) method. The Patient has a history of Graves’ disease and completed an antithyroid drug (ATD) course, the indicator/intervention is low TSH level 4 weeks following ATD withdrawal, the comparison is normal or high TSH level 4 weeks following ATD withdrawal, and the outcome is a recurrence of Graves’ disease.

### Literature search

Literature searching was conducted on PubMed, Scopus, Cochrane, Proquest, EBSCO in August 2019 and Google Scholar in October 2020. The searching strategy was described in Table 1. Our searching was not restricted by year of publication and we excluded non-English articles.

**Table 1:** Searching strategy.

Search engine	Search term
**Pubmed**	(("Thyrotropin"[Mesh]) AND "Graves’ Disease"[Mesh]) AND "Recurrence"[Mesh]
**Scopus**	("Graves’ disease" AND ("Recurrence" OR "Recurrence") AND ("Thyroid Stimulating Hormone" OR "TSH" OR "Thyrotropin") AND ("Prognosis" OR "Prognostic Factor"))
**Cochrane**	Thyrotropin and Graves’ disease and Recurrence and Prognosis
**Proquest**	ab(thyrotropin) AND ab(Graves’ disease) AND ab(recurrence)
**EBSCO**	AB thyrotropin AND AB Graves’ disease AND AB recurrence AND AB prognosis
**Google Scholar**	"thyrotropin" AND "Graves’ disease" AND "recurrence" AND "prognosis"

### Assessment of study eligibility

Two authors independently evaluated study eligibility. Differences and disagreements were resolved by reassessment and discussion. Articles eligible for critical appraisal should meet our qualification criteria as follows: (1) Prognostic studies in prospective or retrospective cohort design and randomized trial in which there is suitable group (untreated control patients) for our systematic review. (2) Adult patients ≥18 years old. (3) Available serum TSH levels measured 4 weeks following ATD withdrawal. We did not limit the length of follow up after 1 to 2 years following ATD withdrawal. (4) Articles should have available full-text. (5) Articles were published in English, in concordant to the authors’ language proficiency.

### Data collection and quality assessment

Three authors independently extracted data from the studies and collected those into a predefined form, including author names, publication year, sample size, study design, normal TSH ranged defined in each study, inclusion and exclusion criteria, follow-up duration, assessments time, and outcome (might be different for each study). Variables that were sought in this study was patient with a history of Graves' disease (defined as hyperthyroidism in lab findings with clinical signs of Graves’ disease), level of serum TSH measured 4 weeks following ATD withdrawal, and recurrence of Graves' disease (defined as low TSH level obtained 4 weeks after ATD withdrawal). Differences and disagreements were resolved by reassessment and discussion with the fourth author.

Three assumptions regarding missing or unclear information were discussed and agreed upon by three authors. First, the cutoff level of serum TSH difference among the included studies and based on the agreement the low TSH is at least 0.5 mIU/L. Second, was associated with the unclear criteria on two studies (Wood et al. and Talbot et. al) and upon agreement the recurrence criteria on these two studies considered as suppressed TSH. Third, some concerns associated with the outcome in the Hoermann et al. study because of more variation groups other than remission and recurrence and the authors reassessed and agreed that this study focused on the relapse percentage between the groups.

Quality of studies and risk of bias were assessed using Revised Cochrane Risk-of-bias Tool for Randomized Trials (RoB 2) for randomized study and Newcastle-Ottawa Scale for non-randomized studies. Three authors independently extracted the data from all studies and rated the quality of the studies and risk of bias in using the respective tool/guidelines. Differences and disagreements were resolved by reassessment and discussion with the fourth author.

## Results and discussion

### Results

**Fig 1:**
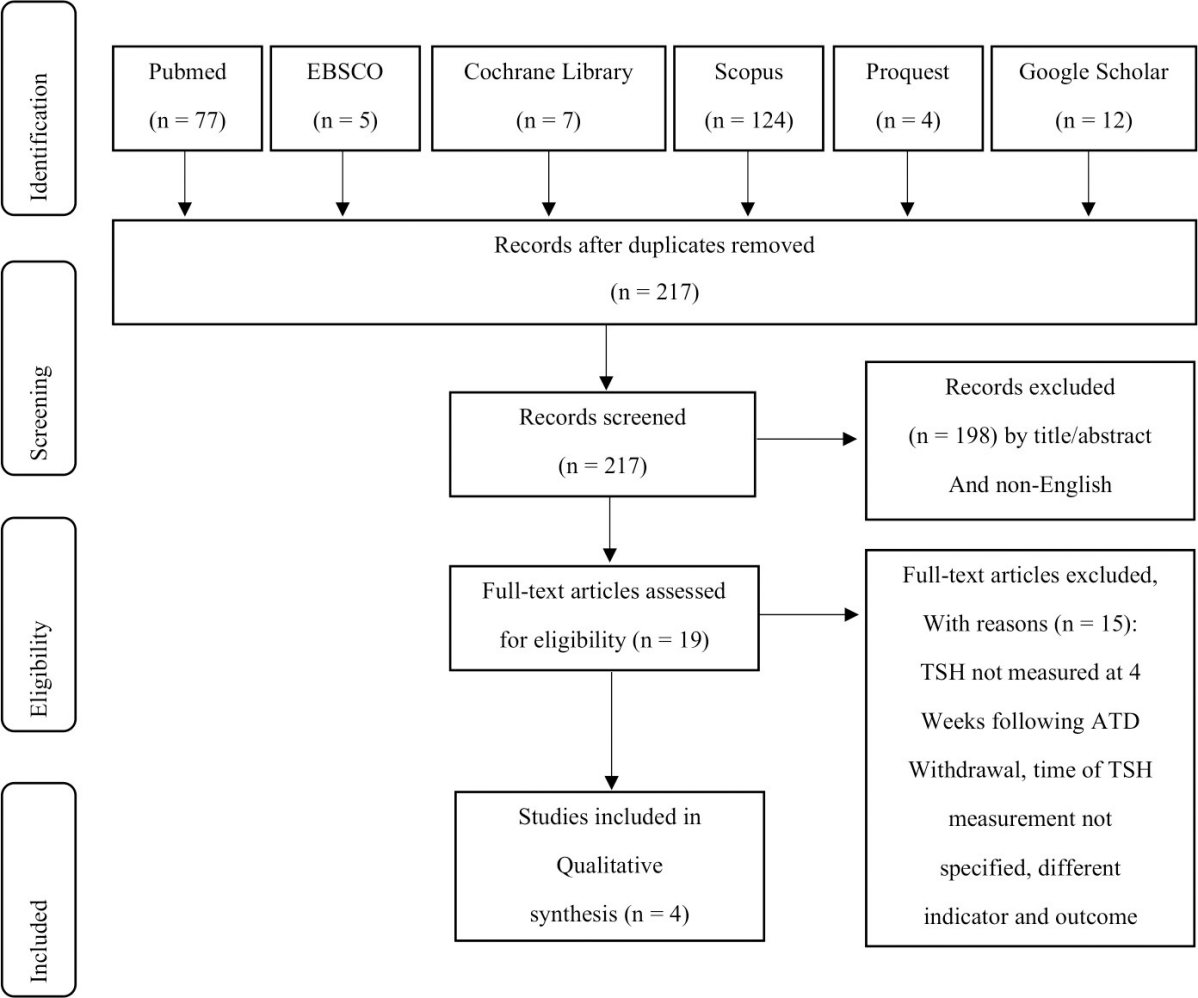
Literature search based on PRISMA flowchart [30].

Literature search retrieved 229 records. (Fig 1). As many as 198 records were excluded by title/abstract and non-English articles, 12 by duplicates, and 15 by either TSH level not measured 4 weeks following ATD withdrawal, time of TSH measurement not specified, or different indicator and outcome. There were 4 articles eligible for this systematic review.

### Study characteristics

Characteristics of the study population, inclusion and exclusion criteria, outcome assessment, and results are shown in Table 2. Of the 4 included studies, 1 was a randomized trial, 2 were prospective cohort studies, while 1 was retrospective cohort study. Hoermann et al. [26], Talbot et al. [19], and Quadbeck et al. [17] used ≤0.3 mIU/L, while Wood et al. [18] used ≤0.13 mU/L as cut-off for low TSH levels. All studies were properly followed up for 1 to 2 years. All studies measured TSH levels at 1 month following treatment withdrawal.

**Table 2:** Characteristics of the selected studies.

References	Year of publication	Sample size	Design	Normal TSH Range	Inclusion criteria	Exclusion criteria	Predictor assessment	Outcome
Quadbeck et al. [17]	2005	96, groups	Prospective cohort, multicenter	0.3–3.0 mIU/L	• Documented diagnosis of GD by clinical signs (orbitopathy) and/or lab findings (thyroxine, TSH, TRAb), imaging (USG or scintigraphy) • Stable euthyroidism for the last 6 months 18–70 years of age	• ATD administration less than 12 or more than 15 months • Hyperthyroidism other than GD • Patients not responding to ATD • Previous thyroid surgery or RAI	Month 1, 3, 6, 12, 18, 24	p <0.01* Se 47% Sp 85% PPV 70% NPV 62%
Hoermann et al. [26]	2002	332, 2 groups	Randomized trial, multicenter	0.3–3.0 mIU/L	• Documented diagnosis of GD by clinical signs (orbitopathy) and/or lab findings (thyroxine, TSH, TRAb), imaging (USG or scintigraphy) • Stable euthyroidism for the last 6 months • 18–70 years of age	• ATD administration less than 12 or more than 15 months • Hyperthyroidism other than GD • Patients not responding to ATD • Previous thyroid surgery or RAI	Month 1, 3, 6, 12, 18, 24	p <0.01* RR 0.43 (95% CI 0.27 to 0.68)
Wood et al. [18]	1995	50, 2 groups	Retrospective cohort, single center	0.13–4.7 mU/L	• Patients with GD and available TSH level 1 month after treatment withdrawal	Hyperthyroidism secondary to multinodular goiter	Month 1, 3, 6, 9, 12	p <0.01*
Talbot et al. [19]	1989	67, 3 groups (Remission, relapse, resistance)	Prospective cohort, single center	0.3–4.5 mIU/L	• Patients with GD • ATD withdrawal when euthyroid state and in minimal Carbimazole for at least 12 months	Not available	Month 1, 3, 6, 9, 12	Non-significant, Sensitivity of low TSH in predicting recurrence 5%

*p <0.01 is considered statistically significant.

Overall, all included studies fulfilled the good validity criteria; all studies included subjects at a common point with sufficient follow-up. The RCT had overall low risk of bias, one cohort study was classified as high-quality study, while the other two were considered as moderate quality study. The studies did not need a blind TSH measurement for laboratory results were considered as the outcome. Quadbeck et al. [17] and Hoermann et al. [26] adjusted important prognostic factors, but the other two study did not.

Quadbeck et al. [17] found that patients with normal TSH level significantly associated with a lower recurrence rate compared to patients with either decreased or increased TSH level (p<0.01; 95% CI 0.8 to 0.12). Hoermann et al. [26] found that higher TSH level had a significant protective effect against recurrence of GD (p<0.01; RR 0.43; 95% CI 0.27 to 0.68). Wood et al. [18] found a significant difference of TSH levels between remission and recurrence group (p<0.01; RR 3.34; 95% CI 0.24 to 0.62). Talbot et al. found that TSH level had low sensitivity (5%) thus making TSH level a poor predictor for the recurrence of GD. They also found the mean of TSH level was 5.6 ± 11.2 mIU/L (95% CI 0.11 to 50). High standard deviation contributed to high standard error thus making a wide confidence interval. Due to narrow confidence interval, all studies except the latter are considered to have good precision in predicting recurrence of GD.

**Fig 2:**
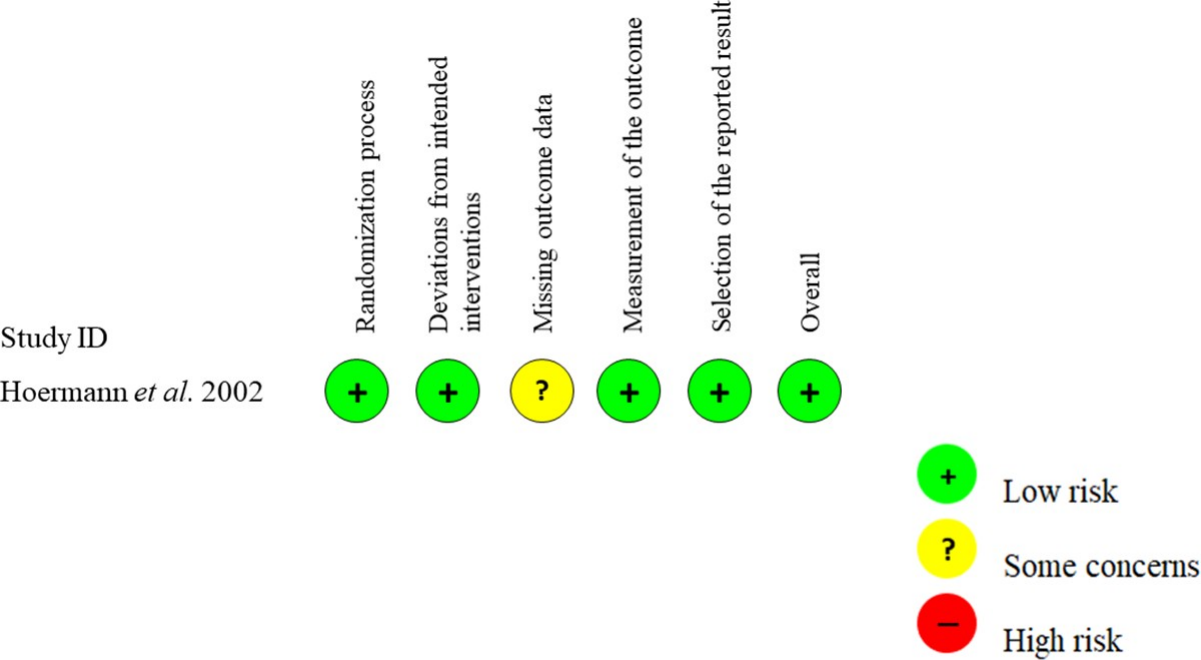
Risk of bias assessment (Revised Cochrane risk-of-bias tool for randomized trials /RoB 2).

All studies can be applied in our daily settings and will make clinically important impacts in predicting the recurrence of GD. One randomized trial study had level of evidence 2b, two prospective cohort studies had level of evidence 1b, and one retrospective cohort study had level of evidence 2b, all according to the Oxford Centre for Evidence-based medicine Guideline [31]. The quality of the studies assessment is presented in Fig 2 and Table 3.

**Table 3:** Risk of bias assessment (Newcastle–Ottawa Quality Assessment Scale criteria).

Study	Selection				Comparability	Outcome			Quality Score
	Representativeness of Exposed Cohort	Selection of the Non-Exposed Cohort	Ascertainment of Exposure	Outcome of Interest	Comparability	Assessment of Outcome	Follow-up Long Enough for Outcome to Occur	Adequacy of Follow-up	
Quadbeck et al. [17]	Prospective cohort, multicenter, 96 (2 groups)*	Yes*	Directly measured from blood sample*	Yes*	The TSH value in the remission group was available for analysis*	Recurrence confirmed by suppressed TSH and elevated fT4 or fT3*	Yes*	100% of subject completed the follow-up *	High
Wood et al. [18]	Retrospective cohort, single-center, 50 (2 groups)	Yes*	Attained from medical record	Yes*	The TSH value in the remission group was available for analysis*	Recurrence criteria is not defined	Yes*	100% of subject completed the follow-up*	Moderate
Talbot et al. [19]	Prospective cohort, single-center (58, 3 groups)	Yes*	Directly measured from blood sample*	Yes*	The TSH value in the remission group was available for analysis*	Recurrence criteria is not defined	Yes*	100% of subject completed the follow-up*	Moderate

High quality: 3 or 4 stars (F) in selection domain AND 1 or 2 stars in comparability domain AND 2 or 3 stars in outcome domain; Moderate quality: 2 stars in selection domain AND 1 or 2 stars in comparability domain AND 2 or 3 stars in outcome/exposure domain; Poor quality: 0 or 1 star in selection domain OR 0 stars in comparability domain OR 0 or 1 stars in outcome/exposure domain.

## Discussion

Two studies were done in prospective cohort and another in retrospective cohort, while one study was a randomized controlled trial. All studies had comparable validity and applicability. Three out of four studies suggested that low TSH level measured 4 weeks following treatment withdrawal was associated with higher risk of disease recurrence. We reviewed four studies on serum TSH level measured 4 weeks following ATD withdrawal as a predictor for GD recurrence. In a randomized trial involving 332 samples, Hoermann et al. [26] observed that 1-year and 2-years recurrence rate of GD in patients in the control group (not given levothyroxine) were 20% and 32% respectively. These rates were significantly lower than those classified as persistent subclinical hyperthyroidism who had significantly low TSH at the beginning of antithyroid drug cessation. Recurrence of hyperthyroidism was shown to be related to serum TSH 4 weeks following treatment withdrawal with RR 0.43 (95% CI 0.27 to 0.68). The mean TSH level for randomized groups was divided into two groups, the control group was 1.1 ± 0.06 mU/L, and 1.1 ± 0.05 mU/L was for the Levothyroxine group. Meanwhile, not eligible for randomization groups were divided into three groups, it was the no-treatment group, the persistence or early relapse group, and the levothyroxine group and the mean TSH level were 0.16 ± 0.03 mU/L, 0.01 ± 0.01 mU/L, and 3.5 ± 0.2 mU/L respectively. They suggested that TSH level is a strong predictor for the first-year recurrence rate of GD with positive, negative predictive value, and accuracy of 56%, 83%, and 75% respectively.

Study from Quadbeck et al. [17] reported that a group of patients with normal TSH levels 4 weeks following ATD withdrawal showed lower recurrence rates at the 2-year follow-up than those with abnormal TSH levels and the difference was statistically significant. Mean TSH level 4 weeks after ATD withdrawal for the remission group was 1.1 ± 0.1 mU/L, the relapse group was 0.8 ± 0.12 mU/L. Low TSH level obtained 4 weeks following ATD withdrawal was found to be a good predictor for recurrence with positive predictive value of 70% with specificity 85%.

Wood et al. [18] also reported that mean TSH levels measured 4 weeks following ATD withdrawal was statistically and significantly lower in recurrence group compared to remission group with both group showed narrow confidence interval. The mean TSH level 4 weeks following ATD withdrawal for the remission group was 1.37 ± 0.82 mU/l and for the recurrence group was 0.43 ± 0.38 mU/l. No patients with TSH level >1.2 mU/L obtained 4 weeks following withdrawal reported of having recurrence in 1-year follow-up. Because the amount of recurrence case in TSH level >1.2 mU/L group was zero, a value of 5 was added in each group to calculate the relative risk. The result showed there was a 3.34-fold increased risk of recurrence in patient with low TSH level measured 4 weeks following ATD withdrawal compared to those with normal TSH level.

In contrast to the three previous studies, Talbot et al. [19] demonstrated that mean TSH levels 4 weeks following ATD withdrawal between remission and recurrence group were not statistically significant. Mean TSH level in remission group was 3.3 mIU/L ± 2.7 and Mean TSH level in recurrence group was 5.6 mIU/L ±11.2 (95% CI of 0.92 to 10.3). The study suggested that TSH level 4 weeks post-treatment was a poor predictor for disease recurrence. The contrast result of this study was likely caused by the patient’s characteristics. The inclusion criteria were unspecific such as clinical diagnosis or signs of GD were not mentioned and also the exclusion criteria were not defined. These characteristics regarding the clinical signs of GD might influence the recurrence or remission of GD.

The four studies have similar sample characteristics and all of them measured TSH level 4 weeks following treatment withdrawal. While cut off level of serum TSH were slightly different from each study, most of the studies agreed that low TSH level is at least 0.5 mIU/L and below. Results from all but study from Talbot et al. are regarded as having good precision and in the study of Wood et al none of the patient who had TSH level >1.2mU/l relapsed during the follow-up period. However, the phenomena look unlikely to happen in the other two latter studies because there were differences or heterogeneity among the study characteristics such as cut-off level of TSH range, TSH measurement kit, patient’s clinical characteristics, and sample size. Though the outcomes of these studies were not reported in the same manner, all but one study concluded that lower TSH level 4 weeks following ATD withdrawal is associated with higher risk of recurrence of Graves’ disease within 1 to 2 years.

Previous studies by Karmisholt et al. and Vos et al. has been conducted in order to predict recurrence in GD patients [12,21]. The Remission Induction and Sustenance in Graves’ Disease (RISG) study in Denmark found that only TRAb measured in the time of diagnosis of GD has the superiority as prognostic marker for GD remission [21]. Other study in the Netherlands successfully created GREAT Score for predicting recurrence in GD. TBII is included as one of the parameters in the score calculation. This score predicts GD recurrence by using recurrence risk (RR), Class I (16%) to Class III (68%) for GREAT Score and Class I+ (4%) to Class IV+ (84%) for GREAT+ Score [12].

This study highlights the utilization of TSH, which is mostly available in many countries. The use of TSH following 4 weeks after ATD withdrawal as predictor for GD recurrence will help clinicians determine the right therapy for each subject. If the subject has higher RR for GD recurrence, other modalities for GD management can be considered.

This study also has several limitations. The first limitation lies on the different management for the subjects after ATD withdrawal. This might affect the incidence of recurrence in each study. Second, the diagnostic method of each study for Graves’ disease is different. Relatively small sample size included in the studies may also affects the validity of this review. Also, our restrictions to English-language publications must also be considered as a limitation. Third, the recurrence criteria were not defined for two of the studies, namely the study of Wood et al. and Talbot et al. The other limitation is the number of studies included in this review is insufficient, and the included literature is outdated. Therefore, it is necessary to carry out further investigations and studies about TSH in predicting GD recurrence, to achieve conclusion in the future.

## Conclusions

In conclusion, three studies showed low TSH level obtained 4 weeks after ATD withdrawal was associated with higher rate of recurrence rate in GD, except in one study. Therefore, TSH level measurement potentially in predicting GD recurrence. Thus, measuring TSH in short period after ATD withdrawal has important clinical implication in predicting short term GD recurrence.

## Supporting information

S1 Checklist PRISMA 2009 checklist—Imam Subekti. (DOC)
